# *rac*-12,14-Dicyclo­propyl-5,8,13,18,21-penta­oxapenta­cyclo­[13.8.0.0^2,11^.0^4,9^.0^17,22^]tricosa-1(15),2(11),3,9(10),16,22(23)-hexa­ene

**DOI:** 10.1107/S1600536811013961

**Published:** 2011-04-22

**Authors:** Viktor A. Tafeenko, Leonid A. Aslanov, Nikolay A. Puretskiy, Aleksander N. Fedotov, Sergei S. Mochalov

**Affiliations:** a Chemistry Department, Moscow State University, 119991 Moscow, Russia

## Abstract

The mol­ecule of the title compound, C_24_H_24_O_5_, has crystallographic twofold symmetry, with the central O atom lying on the rotation axis. The dihedral angle between the best planes of the benzene rings fused to the oxepine fragment is 38.5 (1)°. The dioxine ring adopts a twist form with the ethyl­ene group C atoms deviating by 0.472 (5) and −0.248 (6) Å from the plane defined by the remaining ring atoms.

## Related literature

For details on 2,2′-diacetyl­diphenyl reduction, see: Hall *et al.* (1956 ▶).
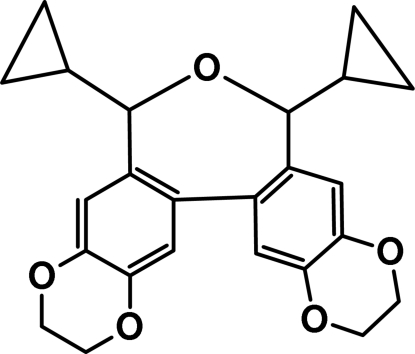


## Experimental

### 

#### Crystal data

C_24_H_24_O_5_
*M* *_r_* = 392.43
Monoclinic, 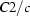

*a* = 14.325 (2) Å
*b* = 7.393 (2) Å
*c* = 19.7260 (12) Å
β = 109.42 (2)°
*V* = 1970.1 (7) Å^3^
*Z* = 4
Ag *K*α radiation
λ = 0.56085 Å
μ = 0.06 mm^−1^
*T* = 295 K
0.10 × 0.05 × 0.05 mm

#### Data collection

Enraf–Nonius CAD-4 diffractometer
1915 measured reflections
1854 independent reflections
966 reflections with *I* > 2σ(*I*)
*R*_int_ = 0.024
2 standard reflections every 120 min  intensity decay: none

#### Refinement

*R*[*F* ^2^ > 2σ(*F* ^2^)] = 0.062
*wR*(*F* ^2^) = 0.170
*S* = 1.01
1855 reflections
133 parameters
H-atom parameters constrained
Δρ_max_ = 0.27 e Å^−3^
Δρ_min_ = −0.18 e Å^−3^

### 

Data collection: *CAD-4 Software* (Enraf–Nonius, 1989 ▶); cell refinement: *CAD-4 Software*; data reduction: *XCAD4* (Harms & Wocadlo, 1995 ▶); program(s) used to solve structure: *SHELXS97* (Sheldrick, 2008 ▶); program(s) used to refine structure: *SHELXL97* (Sheldrick, 2008 ▶); molecular graphics: *DIAMOND* (Brandenburg, 2000 ▶); software used to prepare material for publication: *WinGX* (Farrugia, 1999 ▶).

## Supplementary Material

Crystal structure: contains datablocks I, global. DOI: 10.1107/S1600536811013961/gk2364sup1.cif

Structure factors: contains datablocks I. DOI: 10.1107/S1600536811013961/gk2364Isup2.hkl

Additional supplementary materials:  crystallographic information; 3D view; checkCIF report

## Figures and Tables

**Fig. 1.**
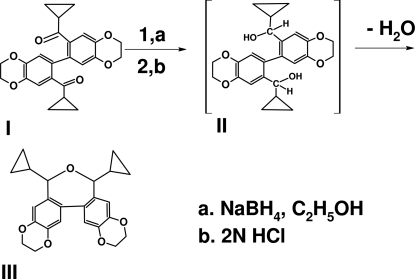
Reduction of (I) did not produce the expected compound (II) but the title compound (III).

**Fig. 2.**
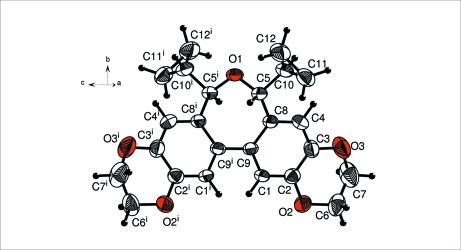
The molecular structure of the title compound, with displacement ellipsoids drawn at the 50% probability level. Atoms C,O and Ci,Oi are related by a twofold axis. Symmetry code: (i) 1 - x, y, 1.5 - z;.

## supplementary crystallographic information

### Comment

In line with our investigations of the intra- and intermolecular transformations of diphenyl derivatives, the results of the transformation of 2,2'-dicyclopropylcarbonyl-bis-ethylenedioxydiphenyl under reduction by NaBH_4_ are presented. Instead of diastereoisomers of 2,2'-bis-cyclopropylhydroxymethyldiphenyls (II) that we might expect based on the Hall's article (Hall *et al.*, 1956) it was shown that the reduction of 2,2'-dicyclopropylcarbonyl-bis-ethylenedioxydiphenyl (I) by NaBH_4_ leads to the formation of 2,3,9,10-bis-ethylenedioxy-5,7-dicyclopropyl-5,7-dihydrodibenz[c,e]- oxepin (III), Fig.1. The ^1^H NMR data of the compound III indicate one of two possible stereoisomeric forms (racemic or *meso*). To determine the structure of the compound III, we carried out an X-ray crystallographic study, which revealed that its structure corresponds to the *erythro* (racemic) form. The dihedral angle between the planes defined by the atoms (Fig.2) C9/C1/C2/C3/C4/C8/C5 (plane 1) and C9^i^/C1^i^/C2^i^/C3^i^/C4^i^/C8^i^/C5^i^ (plane 2) is 41.0 (1)°. Oxygen atom O1 is displaced from the plane 1 by -1.136 (1) and by 1.136 (1) Å from plane 2. The 6-membered dioxine ring adopts a twist conformation, with atoms C6, C7 of the ethylene group displaced from of plane of the remaining dioxine ring atoms by 0.472 (5) and -0.248 (6) Å, respectively. Exept for weak C—H···O interaction between the molecules, no other intermolecular contacts of interest are present.

### Experimental

The reaction scheme is presented in Fig. 1. A mixture of (I) (1.01 g, 2.5 mmol), NaBH_4_ (0.19 g, 5.0 mmol) and 30 ml C_2_H_5_OH was heated (313–323 K) for 48 h and then decomposed with 2 N HCl. The mixture was poured into water (200 ml) and the solid separated, dried and purified by column chromatography. The resulting white precipitate was recrystallized from C_2_H_5_OH.

### Refinement

The positions of all H atoms were determined from Fourier difference maps however for the refinement they were placed in calculated positions and allowed to ride on their parent atoms [C—H = 0.93–0.97 Å] with *U*_iso_(H) = 1.2 *U*_eq_(C).

### Crystal data

**Table:**

C_24_H_24_O_5_	*F*(000) = 832
*M**_r_* = 392.43	*D*_x_ = 1.323 Mg m^−^^3^
Monoclinic, *C*2/*c*	Melting point < 505 K
Hall symbol: -C 2yc	Ag *K*α radiation, λ = 0.56085 Å
*a* = 14.325 (2) Å	Cell parameters from 25 reflections
*b* = 7.393 (2) Å	θ = 11–13°
*c* = 19.7260 (12) Å	µ = 0.06 mm^−^^1^
β = 109.42 (2)°	*T* = 295 K
*V* = 1970.1 (7) Å^3^	Prism, colorless
*Z* = 4	0.10 × 0.05 × 0.05 mm

### Data collection

**Table:**

Enraf–Nonius CAD-4 diffractometer	*R*_int_ = 0.024
Radiation source: fine-focus sealed tube	θ_max_ = 20.0°, θ_min_ = 1.7°
graphite	*h* = −17→16
Non–profiled ω scans	*k* = 0→8
1915 measured reflections	*l* = 0→24
1854 independent reflections	2 standard reflections every 120 min
966 reflections with *I* > 2σ(*I*)	intensity decay: none

### Refinement

**Table:**

Refinement on *F*^2^	Secondary atom site location: difference Fourier map
Least-squares matrix: full	Hydrogen site location: inferred from neighbouring sites
*R*[*F*^2^ > 2σ(*F*^2^)] = 0.062	H-atom parameters constrained
*wR*(*F*^2^) = 0.170	*w* = 1/[σ^2^(*F*_o_^2^) + (0.0788*P*)^2^] where *P* = (*F*_o_^2^ + 2*F*_c_^2^)/3
*S* = 1.01	(Δ/σ)_max_ < 0.001
1855 reflections	Δρ_max_ = 0.27 e Å^−^^3^
133 parameters	Δρ_min_ = −0.18 e Å^−^^3^
0 restraints	Extinction correction: *SHELXL97* (Sheldrick, 2008), Fc^*^=kFc[1+0.001xFc^2^λ^3^/sin(2θ)]^-1/4^
Primary atom site location: structure-invariant direct methods	Extinction coefficient: 0.0097 (18)

### Special details

**Table:**

Geometry. All e.s.d.'s (except the e.s.d. in the dihedral angle between two l.s. planes) are estimated using the full covariance matrix. The cell e.s.d.'s are taken into account individually in the estimation of e.s.d.'s in distances, angles and torsion angles; correlations between e.s.d.'s in cell parameters are only used when they are defined by crystal symmetry. An approximate (isotropic) treatment of cell e.s.d.'s is used for estimating e.s.d.'s involving l.s. planes.
Refinement. Refinement of *F*^2^ against ALL reflections. The weighted *R*-factor *wR* and goodness of fit *S* are based on *F*^2^, conventional *R*-factors *R* are based on *F*, with *F* set to zero for negative *F*^2^. The threshold expression of *F*^2^ > σ(*F*^2^) is used only for calculating *R*-factors(gt) *etc*. and is not relevant to the choice of reflections for refinement. *R*-factors based on *F*^2^ are statistically about twice as large as those based on *F*, and *R*- factors based on ALL data will be even larger.

### Fractional atomic coordinates and isotropic or equivalent isotropic displacement parameters (Å^2^)

**Table:**

	*x*	*y*	*z*	*U*_iso_*/*U*_eq_	
O1	0.5000	0.5604 (4)	0.7500	0.0542 (9)	
O2	0.36252 (17)	−0.1674 (3)	0.56383 (12)	0.0562 (7)	
O3	0.3779 (2)	0.1509 (4)	0.48729 (13)	0.0799 (9)	
C1	0.4376 (2)	−0.0085 (4)	0.67251 (16)	0.0399 (8)	
H1	0.4302	−0.1112	0.6975	0.048*	
C2	0.4042 (2)	−0.0103 (4)	0.59872 (17)	0.0441 (8)	
C3	0.4114 (2)	0.1436 (5)	0.56079 (17)	0.0508 (9)	
C4	0.4576 (2)	0.2947 (5)	0.59835 (18)	0.0504 (9)	
H4	0.4640	0.3970	0.5729	0.061*	
C5	0.5540 (3)	0.4568 (4)	0.71343 (18)	0.0473 (9)	
H5	0.6124	0.4067	0.7502	0.057*	
C6	0.3625 (3)	−0.1719 (6)	0.4920 (2)	0.0745 (13)	
H6A	0.3240	−0.2746	0.4671	0.089*	
H6B	0.4297	−0.1860	0.4918	0.089*	
C7	0.3195 (4)	−0.0022 (7)	0.4542 (2)	0.0971 (17)	
H7A	0.3161	−0.0098	0.4044	0.116*	
H7B	0.2527	0.0126	0.4552	0.116*	
C8	0.4942 (2)	0.2987 (4)	0.67201 (17)	0.0415 (8)	
C9	0.4821 (2)	0.1442 (4)	0.71016 (15)	0.0382 (7)	
C10	0.5896 (3)	0.5892 (4)	0.67094 (19)	0.0540 (9)	
H10	0.5379	0.6569	0.6346	0.065*	
C11	0.6795 (3)	0.5512 (5)	0.6530 (2)	0.0632 (11)	
H11A	0.6813	0.5922	0.6068	0.076*	
H11B	0.7137	0.4381	0.6698	0.076*	
C12	0.6811 (3)	0.6909 (6)	0.7077 (2)	0.0762 (13)	
H12A	0.7163	0.6627	0.7577	0.091*	
H12B	0.6839	0.8168	0.6947	0.091*	

### Atomic displacement parameters (Å^2^)

**Table:**

	*U*^11^	*U*^22^	*U*^33^	*U*^12^	*U*^13^	*U*^23^
O1	0.079 (2)	0.0269 (17)	0.073 (2)	0.000	0.0467 (19)	0.000
O2	0.0672 (15)	0.0504 (15)	0.0497 (14)	−0.0187 (13)	0.0176 (12)	−0.0134 (12)
O3	0.102 (2)	0.083 (2)	0.0439 (16)	−0.0305 (18)	0.0109 (14)	0.0079 (14)
C1	0.0506 (18)	0.0265 (17)	0.0463 (18)	−0.0009 (14)	0.0210 (15)	0.0034 (14)
C2	0.0438 (18)	0.041 (2)	0.0496 (19)	−0.0039 (16)	0.0190 (15)	−0.0035 (16)
C3	0.056 (2)	0.056 (2)	0.040 (2)	−0.0070 (18)	0.0159 (16)	0.0017 (18)
C4	0.062 (2)	0.038 (2)	0.055 (2)	−0.0074 (17)	0.0250 (18)	0.0071 (17)
C5	0.067 (2)	0.0287 (17)	0.054 (2)	−0.0012 (17)	0.0308 (17)	−0.0006 (16)
C6	0.084 (3)	0.088 (3)	0.054 (2)	−0.036 (3)	0.027 (2)	−0.026 (2)
C7	0.112 (4)	0.116 (5)	0.046 (2)	−0.045 (3)	0.003 (2)	0.002 (3)
C8	0.0517 (19)	0.0297 (18)	0.050 (2)	−0.0026 (15)	0.0261 (16)	−0.0006 (15)
C9	0.0441 (17)	0.0272 (16)	0.0469 (17)	−0.0004 (15)	0.0199 (15)	−0.0025 (14)
C10	0.070 (2)	0.0336 (19)	0.064 (2)	0.0011 (18)	0.0299 (19)	0.0089 (17)
C11	0.067 (2)	0.058 (2)	0.075 (3)	0.001 (2)	0.038 (2)	0.014 (2)
C12	0.094 (3)	0.060 (3)	0.073 (3)	−0.035 (2)	0.027 (2)	−0.003 (2)

### Geometric parameters (Å, °)

**Table:**

O1—C5	1.441 (3)	C6—C7	1.484 (6)
O2—C2	1.380 (4)	C6—H6A	0.9700
O2—C6	1.417 (4)	C6—H6B	0.9700
O3—C3	1.368 (4)	C7—H7A	0.9700
O3—C7	1.430 (5)	C7—H7B	0.9700
C1—C2	1.373 (4)	C8—C9	1.410 (4)
C1—C9	1.385 (4)	C9—C9^i^	1.482 (6)
C1—H1	0.9300	C10—C11	1.470 (5)
C2—C3	1.385 (4)	C10—C12	1.475 (5)
C3—C4	1.381 (5)	C10—H10	0.9800
C4—C8	1.371 (4)	C11—C12	1.487 (5)
C4—H4	0.9300	C11—H11A	0.9700
C5—C10	1.486 (4)	C11—H11B	0.9700
C5—C8	1.517 (4)	C12—H12A	0.9700
C5—H5	0.9800	C12—H12B	0.9700
			
C5^i^—O1—C5	115.7 (3)	C6—C7—H7A	109.4
C2—O2—C6	112.0 (3)	O3—C7—H7B	109.4
C3—O3—C7	113.4 (3)	C6—C7—H7B	109.4
C2—C1—C9	120.8 (3)	H7A—C7—H7B	108.0
C2—C1—H1	119.6	C4—C8—C9	118.5 (3)
C9—C1—H1	119.6	C4—C8—C5	122.4 (3)
C1—C2—O2	118.5 (3)	C9—C8—C5	119.0 (3)
C1—C2—C3	120.2 (3)	C1—C9—C8	119.4 (3)
O2—C2—C3	121.3 (3)	C1—C9—C9^i^	120.3 (2)
O3—C3—C4	118.2 (3)	C8—C9—C9^i^	120.3 (2)
O3—C3—C2	122.8 (3)	C11—C10—C12	60.7 (2)
C4—C3—C2	118.9 (3)	C11—C10—C5	120.2 (3)
C8—C4—C3	122.2 (3)	C12—C10—C5	118.3 (3)
C8—C4—H4	118.9	C11—C10—H10	115.5
C3—C4—H4	118.9	C12—C10—H10	115.5
O1—C5—C10	105.9 (2)	C5—C10—H10	115.5
O1—C5—C8	112.2 (2)	C10—C11—C12	59.8 (2)
C10—C5—C8	116.2 (3)	C10—C11—H11A	117.8
O1—C5—H5	107.3	C12—C11—H11A	117.8
C10—C5—H5	107.3	C10—C11—H11B	117.8
C8—C5—H5	107.3	C12—C11—H11B	117.8
O2—C6—C7	110.1 (4)	H11A—C11—H11B	114.9
O2—C6—H6A	109.6	C10—C12—C11	59.5 (2)
C7—C6—H6A	109.6	C10—C12—H12A	117.8
O2—C6—H6B	109.6	C11—C12—H12A	117.8
C7—C6—H6B	109.6	C10—C12—H12B	117.8
H6A—C6—H6B	108.1	C11—C12—H12B	117.8
O3—C7—C6	111.0 (3)	H12A—C12—H12B	115.0
O3—C7—H7A	109.4		

Symmetry codes: (i) −*x*+1, *y*, −*z*+3/2.
